# Impact of Self-Monitoring Blood Glucose on Glycaemic Control Among Insulin-Treated Patients With Diabetes Mellitus in Northeastern Tanzania: A Randomised Controlled Trial

**DOI:** 10.1155/2024/6789672

**Published:** 2024-06-10

**Authors:** Sophia S. Muhali, Fatma S. Muhali, Sayoki G. Mfinanga, Abid M. Sadiq, Annette A. Marandu, Norman J. Kyala, Fuad H. Said, Eliada B. Nziku, Tumaini E. Mirai, James S. Ngocho, Henry L. Mlay, Gilbert G. Waria, Angelina Chambega, Stella N. Kessy, Kajiru G. Kilonzo, Furaha S. Lyamuya, Elifuraha W. Mkwizu, Elichilia R. Shao, Nyasatu G. Chamba

**Affiliations:** ^1^ Faculty of Medicine Kilimanjaro Christian Medical University College, Moshi, Tanzania; ^2^ Department of Endocrinology Muhimbili National Hospital, Dar es Salaam, Tanzania; ^3^ National Institute for Medical Research Muhimbili Research Centre, Dar es Salaam, Tanzania; ^4^ Department of Internal Medicine Kilimanjaro Christian Medical Centre, Moshi, Tanzania; ^5^ Institute of Public Health Kilimanjaro Christian Medical University College, Moshi, Tanzania; ^6^ Nutrition Unit Kilimanjaro Christian Medical Centre, Moshi, Tanzania

**Keywords:** diabetes mellitus, glycaemic control, glycated haemoglobin, self-monitoring blood glucose, Tanzania

## Abstract

**Introduction:** Tracking of blood glucose levels by patients and care providers remains an integral component in the management of diabetes mellitus (DM). Evidence, primarily from high-income countries, has illustrated the effectiveness of self-monitoring of blood glucose (SMBG) in controlling DM. However, there is limited data on the feasibility and impact of SMBG among patients in the rural regions of sub-Saharan Africa. This study is aimed at assessing SMBG, its adherence, and associated factors on the effect of glycaemic control among insulin-treated patients with DM in northeastern Tanzania.

**Materials and Methods:** This was a single-blinded, randomised clinical trial conducted from December 2022 to May 2023. The study included patients with DM who had already been on insulin treatment for at least 3 months. A total of 85 participants were recruited into the study and categorised into the intervention and control groups by a simple randomization method using numbered envelopes. The intervention group received glucose metres, test strips, logbooks, and extensive SMBG training. The control group received the usual care at the outpatient clinic. Each participant was followed for a period of 12 weeks, with glycated haemoglobin (HbA_1c_) and fasting blood glucose (FBG) being checked both at the beginning and at the end of the study follow-up. The primary and secondary outcomes were adherence to the SMBG schedule, barriers associated with the use of SMBG, and the ability to self-manage DM, logbook data recording, and change in HbA_1c_. The analysis included descriptive statistics, paired *t*-tests, and logistic regression.

**Results:** Eighty participants were analysed: 39 in the intervention group and 41 in the control group. In the intervention group, 24 (61.5%) of patients displayed favourable adherence to SMBG, as evidenced by tests documented in the logbooks and glucometer readings. Education on SMBG was significantly associated with adherence. Structured SMBG improved glycaemic control with a HbA_1c_ reduction of −1.01 (95% confidence interval (CI) −1.39, −0.63) in the intervention group within 3 months from baseline compared to controls of 0.18 (95% CI −0.07, 0.44) (*p* < 0.001).

**Conclusion:** Structured SMBG positively impacted glycaemic control among insulin-treated patients with DM in the outpatient clinic. The results suggest that implementing a structured testing programme can lead to significant reductions in HbA_1c_ and FBG levels.

**Trial Registration:** Pan African Clinical Trials Registry identifier: PACTR202402642155729.

## 1. Introduction

Diabetes mellitus (DM) poses a considerable socioeconomic burden due to its high prevalence and impact on morbidity and mortality, especially from cardiovascular and neuropathic complications [1]. Globally, DM affects a large number of adults, with approximately one in two cases remaining undiagnosed [2]. According to the International Diabetes Federation (IDF) diabetes atlas, the African region faces its own mounting challenges, with an estimated 24 million adults aged 20–79 living with DM, which is projected to increase to 33 million by 2030 and 55 million by 2045 [3]. Tanzania, a country within the IDF African region, contends with a DM prevalence of 12.3% and an estimated expenditure of USD 149.8 per person on managing the disease [3]. In Kilimanjaro, a northeastern Tanzanian region, the prevalence of DM is approximately 5.7% [4].

Effective management of DM necessitates a multifaceted approach, including lifestyle modifications, medication self-administration, and self-monitoring of blood glucose (SMBG). SMBG is a valuable tool that empowers patients on insulin therapy to gauge the effects of lifestyle changes and medications on their blood glucose levels [5]. Structured SMBG is an approach that involves gathering blood glucose readings following a specific schedule, interpreting them, and using the information to make suitable pharmacological or lifestyle modifications [6]. Current evidence underscores the crucial role of SMBG in DM management, which not only involves self-measurement but also the interpretation of blood glucose readings and appropriate responses. This contributes to the reduction of disease progression and the prevention of serious complications [7, 8].

Considering the diurnal variations in glucose levels, structured SMBG has proven valuable in assessing these fluctuations independently of glycated haemoglobin (HbA_1c_) measurements [9]. Periodic evaluation of SMBG readings is crucial to ensuring alignment with HbA_1c_ measurements [10]. However, previous studies conducted in both developed and developing countries have reported suboptimal rates of SMBG adherence, reflecting a pressing issue in DM management [11].

Recent research has indicated that structured SMBG regimens can lead to improved clinical outcomes, particularly in insulin-treated patients with type 2 DM (T2DM) [12]. Notably, leading diabetes organisations, such as the American Diabetes Association and the National Institute for Health and Care Excellence, provide guidelines for SMBG frequency and interpretation to optimise diabetes management. Guidelines recommend regular assessment and review of SMBG diaries and HbA_1c_ levels, as well as appropriate treatment adjustments until patients achieve their target HbA_1c_ levels [13].

In Tanzania, the national standard treatment guideline suggests SMBG for patients on insulin and glucose-lowering agents that may cause hypoglycaemia, but it lacks clear guidance regarding the frequency and structure of SMBG performance [14]. Interestingly, patients with DM tend to monitor their blood glucose less frequently than recommended by their healthcare providers. While studies from other parts of sub-Saharan Africa have provided some insights into SMBG practices [15], there is a conspicuous gap in knowledge within Tanzania, warranting further exploration. This study aimed at assessing SMBG, its adherence, and associated factors on the effect of glycaemic control among insulin-treated patients with DM at a zonal hospital in northeastern Tanzania.

## 2. Materials and Methods

### 2.1. Study Design

This was a hospital-based interventional study, a 6-month randomized clinical trial that involved all eligible patients with DM who were enrolled to evaluate the impact of structured SMBG among insulin-dependent patients. It was conducted at the outpatient diabetes clinic at Kilimanjaro Christian Medical Centre (KCMC) from December 2022 to May 2023. Patients were enrolled consecutively based on eligibility criteria and then followed up for 3 months from baseline. This study included adult patients with DM who were on insulin treatment at least 3 months before enrolment, regardless of whether it was in addition to oral hypoglycaemic medications or not.

In this study, the inclusion criteria encompassed patients with DM aged 18 years or older, diagnosed with either Type 1 DM (T1DM) or T2DM, and currently receiving insulin treatment for a minimum of 3 months prior to enrolment. Additionally, eligible participants had HbA_1c_ levels exceeding ≥ 8%, were capable of reading or writing, or had a dependable guardian who could assist in this regard. Conversely, the exclusion criteria excluded patients with DM with HbA_1c_ levels exceeding ≥ 8% who were pregnant, those with a glomerular filtration rate below 15 ml/min, and individuals with haemoglobin levels falling below 9 g/dL.

Based on existing literature, this study expected a HbA_1c_ change of 1.2% in the intervention group and 0.2% in the control group, with a standard deviation (SD) of 2 or less [16]. A significance level (*Z* constant) of 0.05 was used. Taking into account an attrition rate of 10%, this study determined that a sample size of 84 patients with DM would be appropriate for the study, generating a power of above 80%.

### 2.2. Enrolment Procedure

Identification of participants was done through the Electronic Health Management System and through the diabetes clinic attendance registry. Patients were called to attend clinics before the due date in order to further assess their eligibility. At the beginning of the study, participants were checked for eligibility and then enrolled in the study within 5 days of eligibility checking. Participant demographics and laboratory findings, including baseline HbA_1c_, were done at the beginning of the study. If eligible, participants were then recruited for the study and followed up for 12 weeks. A simple randomization method was used to randomise participants into two arms: the intervention and control arms. Random numbers were assigned to participants using envelopes, employing a computer-generated method. The randomization was a 1:1 method used by the computer to put patients in either group, as the participants and caregivers were blinded, making it a single-blinded randomization study. An average of four to six patients with DM were recruited during clinic visits.

Demographic data and basic clinical characteristics, including DM self-management and awareness of glycaemic targets, were assessed using a closed-ended questionnaire. The questionnaire was adapted from the Diabetes Self-Management Questionnaire-Revised [17] and the Personal Diabetes Questionnaire [18]. The variables included were as follows: age, classified into 18–34 years, 35–59 years, or > 60 years; occupation, classified as employed or unemployed; marital status, classified as married or single (including those separated or widowed); sex, classified as male or female; education level, classified as primary/secondary or higher; and body mass index (BMI), classified as underweight (< 18.5 kg/m^2^), normal (18.5–24.9 kg/m^2^), overweight (25.0–29.9 kg/m^2^), or obese (≥ 30.0 kg/m^2^). The duration of an insulin regimen was classified as < 1 year, 1–5 years, 5–10 years, or > 10 years. Dosage frequency was grouped into once daily, twice daily, or thrice daily. The duration of diabetes was divided into three categories: 1–5 years, 5–10 years, and > 10 years. Additional behavioural factors like education on SMBG, insulin dose adjustment, challenges in blood glucose measurement, and awareness of glycaemic targets were dichotomized as yes or no. HbA_1c_ tests were conducted during enrolment and at the 3-month mark using the Diabetes Control and Complications Trial (DCCT)-aligned Finecare™ FIA Meter Plus analyser, which was regularly calibrated. This machine is recognized by the National Glycohemoglobin Standardization Program (NGSP) for assessing HbA_1c_ and is, thus, calculated according to NGSP standards. Additionally, fasting blood glucose (FBG) levels were measured at each visit using a finger stick and the GlucoNavii glucometer.

### 2.3. Data Collection Procedure

The individuals who were trained for data collection were diabetic nurses, nutritionists, and medical doctors. The study involved two patient groups: the SMBG intervention arm and the control arm. In the structured SMBG intervention arm, participants received comprehensive education on DM self-management and used a structured staggered SMBG approach. This was an alternating testing regimen that created blood glucose profiles that necessitated testing before and 2 hours after breakfast on Day 1, before and 2 hours after lunch on Day 2, and before and 2 hours after supper on Day 3, and subsequently over the week. They were equipped with glucometers, test strips, and logbooks to monitor their blood glucose levels daily. The logbook contained pre- and postmeal readings, along with extra readings in cases of hypoglycaemia, which were recorded by either the caregiver or the patient. These entries were cross-referenced with glucometer readings, with adherence defined as a minimum of 150 readings over 12 weeks. These individuals attended an extra study visit for additional SMBG education and training, which included insulin dosage adjustments. The training emphasized the correct methods of measuring and recording blood glucose levels at various times of the day. They were instructed to monitor their FBG levels, among other readings, throughout the week and perform a blood glucose profile on the 7th day of the week. Regular glucometer calibration and reviews of recorded data were part of the process. Nutritional counselling and education on the self-intensification of insulin treatment were provided during monthly visits. Random blood glucose and FBG were assessed using glucometers, with participants fasting for 8 hours before testing, and they had access to the study coordinator's contact information for questions and follow-up. In the control arm, participants continued to receive standard DM care, which included routine testing and monthly follow-up consultations involving FBG checks, medication prescriptions, and HbA_1c_ measurements at baseline and 3 months later in the study.

### 2.4. Outcome Measures

The primary endpoint was the reduction of HbA_1c_ by at least 1.2% from baseline. The secondary endpoint was the adherence to structured SMBG and its acceptability, as well as the facilitators and barriers associated with the use of SMBG and the ability to self-manage DM. Adherence to SMBG was calculated by dividing the number of tests actually performed by the number of tests that were supposed to be performed. Adherence of 80% or higher was considered good adherence.

### 2.5. Statistical Analysis

The Stata version 15 software was used to perform the analysis. Descriptive statistics were used, involving frequency and percentages for categorical variables and mean (SD) or median (IQR) for numerical variables. Statistical tests like the Pearson chi-square test or Fisher's exact test were employed to analyse the relationship between SMBG and sociodemographic factors postintervention and also between SMBG and behavioural factors in insulin-treated patients with DM before the intervention. A paired *t*-test evaluated changes in mean HbA_1c_ within both the intervention and control groups postintervention. Additionally, a two-sample *t*-test compared mean HbA_1c_ levels between the intervention and control groups before and after the intervention. Logistic regression was used to determine the association between SMBG and behavioural factors, providing odds ratios (OR) and 95% confidence intervals (CIs). Analysis of covariates (ANCOVA) determined the independent effect of the intervention on glycaemic control. Model selection was based on the Akaike information criterion (AIC), with the smallest AIC considered the best fit.

## 3. Results

**Figure 1:**
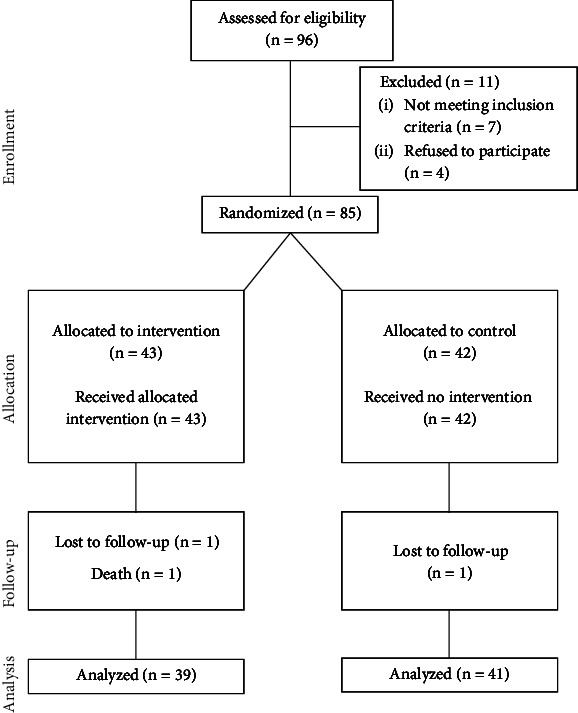
Flow chart for selection of study participants.

Out of the 96 patients initially recruited from the DM clinic, 89 met the inclusion criteria, and 85 were randomized, with 43 in the intervention group and 42 in the control group. After 3 months, four patients were lost to follow-up, three from the intervention arm and one from the control arm. Unfortunately, one patient from the intervention group passed away due to a respiratory illness, leaving a total of 80 study participants for analysis (Figure 1).

About half of the participants (51.3%) in the intervention group were within the age range of 35–59 years, while participants aged > 60 years constituted a larger proportion (65.9%) in the control group. The majority of the participants were female in both the intervention and control groups, with 24 (61.5%) and 33 (80.5%), respectively. Both the intervention group (64.1%) and the control group (65.9%) had a similar proportion of married individuals. Most of the participants were unemployed (intervention group, 51.3% vs. control group, 75.6%). Occupation also exhibited a significant difference, with a higher proportion of employed participants in the intervention group 19 (48.7%) compared to the control group 10 (24.4%).

The intervention group had a median total insulin dose of 48 units (IQR 36–64) per day, compared to 55 units (IQR 36–72) in the control group. For the intervention and control groups, the mean waist circumference was 93.9 cm (SD 12.9) and 95 cm (SD 13.2), respectively. In both groups, most participants were diagnosed with DM for more than 10 years and were on insulin treatment for more than 1 year (Table 1).

There was a postintervention reduction of HbA_1c_ within the intervention group of −1.01 (CI −1.39, −0.63) and a 0.18 (CI −0.07, 0.44) reduction in the control group, whereby the mean difference in the intervention group was statistically significant (*p* < 0.001) (Table 2). While studying the differences between the two groups, the mean HbA_1c_ in the intervention and control groups preintervention was 11.1% and 10.7%, respectively. The mean difference in HbA_1c_ between the two study arms was 0.39 (CI −0.42, 1.21), which was statistically insignificant during the commencement of the study but rendered a significant difference of −0.806 (CI −1.59, −0.02) at the end of the study. Similarly, FBG preintervention was 8.91 mmol/L and 10.23 mmol/L in the intervention and control groups, respectively, with the postintervention FBG being 6.85 mmol/L in the intervention group and 8.55 mmol/L in the control group, respectively. This was a −1.69 (CI −2.68, −0.70) difference, showing statistical significance in FBG between the two groups (Table 3).

**Figure 2:**
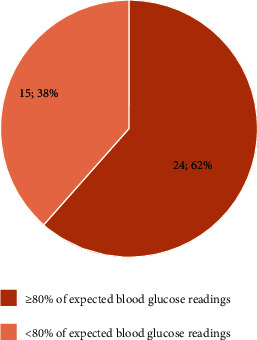
Adherence to SMBG among the intervention group (*n* = 39).

Among the intervention group, the study found that the majority of participants (62%) demonstrated good adherence to SMBG based on logbook records, whereas 38% had poor adherence with fewer blood glucose readings (Figure 2). Social demographic characteristics such as age, education level, occupation, marital status, and BMI did not appear to significantly influence SMBG adherence in the intervention group. However, there was a potential, albeit statistically insignificant, association between the duration of insulin prescription and SMBG adherence (*p* = 0.096). Interestingly, participants with a longer history of insulin use tended to exhibit lower adherence rates (46.7%) (Table 4).

The analysis showed being taught SMBG was significantly associated with self-monitoring (*p* = 0.01) whereby 33 (55.9%) of participants who had ever been taught SMBG performed it while 26 (44.1%) did not (Table S1). After adjusting for potential confounders, individuals in the intervention group had a decrease in HbA_1c_ levels by 1.14 units compared to the control group (adjusted coefficients −1.14 (95% CI: −1.91 to −0.36; *p* = 0.004). Also, participants with secondary education and above had an increase in HbA_1c_ levels by 1.21 units compared to participants with primary education only adjusting for other factors (adjusted coefficients 1.21 (95% CI: 0.43–1.99; *p* = 0.003) (Table S2).

## 4. Discussion

The findings in this study demonstrated the effectiveness of the SMBG intervention in improving glycaemic control. The group that received structured SMBG training and implemented SMBG had achieved better HbA_1c_ after 3 months of follow-up in comparison to the group with no SMBG implementation.

The study also revealed that more than half of the participants in the intervention group demonstrated good adherence to SMBG based on logbook records. These findings align with studies conducted in various countries, such as Australia, Italy, and Rwanda [19, 20], which have reported relatively higher levels of adherence to SMBG. However, there was still a significant proportion of patients who had poor adherence, with less than 80% of expected blood glucose readings, thus causing suboptimal adherence levels. It is important to note that studies conducted in Malaysia, Nigeria, and East Africa (specifically Kenya) [15, 21, 22] have also emphasized the difficulties in maintaining SMBG adherence in their respective settings, thus highlighting the challenges in achieving consistent adherence to SMBG. Unlike these studies, which largely relied on self-reported SMBG data from urban and semiurban populations, this study implemented a more structured approach to SMBG with detailed information recorded in logbooks.

This study also provided extensive training to the group responsible for implementing SMBG. The importance of structured SMBG methods and the role of education in encouraging adherence were made apparent through this research. Moreover, in this study, participants were from both rural and urban populations, in comparison to a study in Nigeria that showed adherence to SMBG was less than 10% in the rural population [22]. The diversity of the participants in this study highlights the influence of access to healthcare resources on adherence levels. The variation in adherence patterns among different populations emphasizes the role of socioeconomic factors. Although adherence was still suboptimal, another interventional approach in this study was the provision of strips and glucometers to the participants. This differs from other studies where participants had to purchase these materials, thus demonstrating the role of resource availability in SMBG adherence. This indicates that adherence to SMBG can vary across different populations and healthcare systems and suggests that awareness alone does not guarantee adherence to SMBG. This also demonstrates the need for further investigation into the factors influencing SMBG adherence in different healthcare settings.

The study also demonstrated the effectiveness of the SMBG intervention in improving glycaemic control. Similar positive effects of structured SMBG on glycaemic control were observed in studies done in Serbia, Japan, and European and Middle Eastern countries [12, 23]. Similarly, a study done in Rwanda also showed significant improvement of HbA_1c_ within 3 months of SMBG intervention [19]. However, the relationship between SMBG and glycaemic control is not always uniform across different studies. For instance, a study in Tokyo yielded results contrasting with those of this study, as it did not observe improvements in glycaemic control within the initial 3 months of structured SMBG but noted positive changes after a 6-month period [24]. The differences in these findings may be attributed to variations in how glycaemic control was defined and measured. In the Tokyo study, it was defined as the normalization of HbA_1c_ to less than 6.9%, whereas this study considered any drop of HbA_1c_ by at least 1.1% from the baseline as significant in comparison to the control group. Moreover, a retrospective study conducted in Kenya did not find a correlation between SMBG adherence and glycaemic control, although there was a reduction in HbA_1c_ levels from baseline [15]. While the study did report a reduction in HbA_1c_ levels from baseline, the lack of a clear link between SMBG and glycaemic control was not attained. These inconsistencies may be attributed to various factors, including differences in study design and participant characteristics.

Therefore, the study provides evidence that structured SMBG has a positive impact on glycaemic control among insulin-treated outpatient diabetes clinic patients in northeastern Tanzania. The results suggest that implementing a structured testing program can lead to significant reductions in HbA_1c_ and FBG levels. Furthermore, the study underscores the positive impact of structured SMBG on glycaemic control. It aligns with previous research that has shown significant reductions in HbA_1c_ levels with the implementation of a structured testing program. Additionally, while demographic factors may not be major determinants of SMBG adherence, the duration of insulin therapy might play a subtle role in influencing adherence levels. However, other factors such as age, occupation, comorbidities, BMI, insulin regimen, type of insulin prescription, and insulin dose did not significantly affect glycaemic control in this study.

The study had some limitations, including the relatively short study duration, which restricted the assessment of the long-term sustainability of SMBG practices and their effects on glycaemic control. A longer follow-up would have provided insights into the intervention's durability. Secondly, the study's confinement to a single outpatient clinic may limit the generalizability of its findings to other healthcare settings and populations. Including multiple centres in future studies would enhance external validity. Furthermore, there is the potential for selection bias, as participants were recruited from a single clinic, introducing biases in the sample. Differences in characteristics and motivations between participants and nonparticipants could impact the study results. The reliance on self-reported data, particularly logbook records, is another limitation. This method is vulnerable to recall bias and inaccuracies, potentially leading to misclassification of adherence levels. Exploring alternative data collection methods may mitigate these issues. Thirdly, this study did not look into the factors contributing to nonadherence to SMBG; hence, further research is advised. Lastly, the study lacked qualitative data, which could have provided a deeper understanding of the barriers and facilitators to self-monitoring behaviour. Incorporating qualitative data could enhance comprehension of SMBG adherence in greater detail.

## 5. Conclusion

This study highlights SMBG's efficacy in improving glycaemic control, as evidenced by a significant reduction in HbA_1c_ levels and FBG levels within the intervention group. It underscores the challenges of SMBG in insulin-treated patients with DM, emphasizing the role of education and healthcare providers in fostering proper implementation. This study supports structured SMBG as a valuable tool for glycaemic control, underscoring the importance of education, follow-up, and ongoing research to optimize practices and address adherence barriers. It is recommended to enhance SMBG practices among insulin-treated patients with DM, tailor education for individual needs, and continually evaluate its effectiveness. A future qualitative study should explore patient perspectives on SMBG to understand barriers and facilitators, guiding interventions for improved adherence. Large-scale trials are needed to assess the sustainability and impact of SMBG, and the development of a Tanzania-focused SMBG guideline is recommended. Additionally, offering clear testing recommendations and ensuring glucometer strip availability support SMBG practices.

## Figures and Tables

**Table 1:** Sociodemographic and clinical characteristics of the participants (*n* = 80).

**Variables**	**Intervention (** **n** = 39**)**	**Control (** **n** = 41**)**	**p** **value**
	**n** **(%)**	**n** **(%)**	
Age (years)			0.081
18–34	2 (5.1)	3 (7.3)	
35–59	20 (51.3)	11 (26.8)	
> 60	17 (43.6)	27 (65.9)	
Gender			
Male	15 (38.5)	8 (19.5)	0.061
Female	24 (61.5)	33 (80.5)	
Educational status			0.041
Primary education	13 (33.3)	23 (56.1)	
Secondary and above	26 (66.7)	18 (43.9)	
Occupation			0.024
Employed	19 (48.7)	10 (24.4)	
Unemployed	20 (51.3)	31 (75.6)	
Marital status			0.871
Married	25 (64.1)	27 (65.9)	
Single/separated/widowed	14 (35.9)	14 (34.1)	
BMI (kg/m^2^)			0.605
Underweight	3 (7.7)	1 (2.4)	
Normal	8 (20.5)	8 (19.5)	
Overweight	12 (30.8)	17 (41.5)	
Obese	16 (41.0)	15 (36.6)	
Waist circumference (cm); *mean (SD)*	*93.91 (12.9)*	*95 (13.2)*	
Duration of DM (years)			0.643
1–5	4 (10.3)	2 (4.9)	
6–10	7 (17.9)	7 (17.1)	
> 10	28 (71.8)	32 (78.0)	
Duration on insulin (years)			0.064
< 1	11 (28.2)	3 (7.3)	
1–5	10 (25.6)	16 (39.0)	
6–10	7 (17.9)	12 (29.3)	
> 10	11 (28.2)	10 (24.4)	
Type of insulin			0.840
Premixed insulin	11 (28.2)	14 (34.2)	
Regular + NPH insulin	20 (51.3)	19 (46.3)	
Insulin + oral drugs	8 (20.5)	8 (19.5)	
Total insulin dose (IU); *median (IQR)*	48 (36–64)	55 (36–72)	

**Table 2:** Mean differences between HbA_1c_ at 3 months and baseline within study arms.

**Study arms**	**HbA_1C_ (baseline)** **Mean (SD)**	**HbA_1C_ (3 months)** **Mean (SD)**	**Mean (CI) difference**	**p** **value**
Intervention (*n* = 39)	11.1 (1.64)	10.1 (1.62)	−1.01 (−1.39, −0.63)	<0.001
Control (*n* = 41)	10.7 (1.87)	10.9 (1.90)	0.18 (−0.07, 0.44)	0.140

*Note:* Intervention (*n* = 43 at baseline and *n* = 39 at 3 months); control (*n* = 42 at baseline and *n* = 41 at 3 months).

**Table 3:** Change in HbA_1c_ and FBG of the intervention and control groups.

**Variables**	**Intervention**	**Control**	**Mean (CI) difference**	**p** **value**
	**Mean (±SD)**	**Mean (±SD)**		
Preintervention				
FBG (mmol/L)	8.91 (3.51)	10.23 (4.2)	−1.32 (−3.07, 0.40)	0.134
HbA_1c_ (%)	11.13 (1.81)	10.74 (1.87)	0.39 (−0.42, 1.21)	0.340
Postintervention				
FBG (mmol/L)	6.85 (1.89)	8.55 (2.47)	−1.69 (−2.68, −0.70)	0.001
HbA_1c_ (%)	10.12 (1.62)	10.93 (1.90)	−0.806 (−1.59, −0.02)	0.040

**Table 4:** Association between adherence and participant characteristics in the intervention group (*n* = 39).

**Variable**	**≥ 80% expected blood glucose readings (%)**	**< 80% expected blood glucose readings (%)**	**p** **value**
Age (years)			0.390
18–34	2 (8.3)	0 (0)	
35–59	13 (54.2)	7 (46.7)	
> 60	9 (37.5)	8 (53.3)	
Gender			0.405
Male	8 (33.3)	7 (46.7)	
Female	16 (66.7)	8 (53.3)	
Education level			1.000
Primary education	8 (33.3)	5 (33.3)	
Secondary and above	16 (66.7)	10 (66.7)	
Occupation			0.839
Employed	12 (50.0)	7 (46.7)	
Unemployed	12 (50.0)	8 (53.3)	
Marital status			0.792
Married	15 (62.5)	10 (66.7)	
Single/separated/widowed	9 (37.5)	5 (33.3)	
BMI			0.786
Underweight	2 (8.3)	1 (6.7)	
Normal	5 (20.8)	3 (20.0)	
Overweight	6 (25.0)	6 (40.0)	
Obese	11 (45.8)	5 (33.3)	
Duration on insulin (years)			0.096
< 1	6 (25.0)	5 (33.3)	
1–5	8 (33.3)	2 (13.3)	
6–10	6 (25.0)	1 (6.7)	
> 10	4 (16.7)	7 (46.7)	

## Data Availability

The data that support the findings of this study are available from the corresponding author upon reasonable request.

## References

[1] Vidal Florc M., Jansà Morató M., Galindo Rubio M., Penalba Martínez M. (2018). Factors associated to adherence to blood glucose self-monitoring in patients with diabetes treated with insulin. The dapa study. *Endocrinología, Diabetes y Nutrición (English ed.)*.

[2] Beagley J., Guariguata L., Weil C., Motala A. A. (2014). Global estimates of undiagnosed diabetes in adults. *Diabetes Research and Clinical Practice*.

[3] International Diabetes Federation (2021). *IDF Diabetes Atlas. IDF*.

[4] Stanifer J. W., Cleland C. R., Makuka G. J. (2016). Prevalence, risk factors, and complications of diabetes in the Kilimanjaro region: a population-based study from Tanzania. *PLoS One*.

[5] Ong W. M., Chua S. S., Ng C. J. (2014). Barriers and facilitators to self-monitoring of blood glucose in people with type 2 diabetes using insulin: a qualitative study. *Patient Preference and Adherence*.

[6] Parkin C. G., Buskirk A., Hinnen D. A., Axel-Schweitzer M. (2012). Results that matter: structured vs. unstructured self-monitoring of blood glucose in type 2 diabetes. *Diabetes Research and Clinical Practice*.

[7] Saragoni S., Perrone V., Buda S. (2013). Analysis of clinical outcome and healthcare resource use in insulin treated diabetic patients based on self-monitoring of blood glucose levels. *International Journal of Diabetology & Vascular Disease Research*.

[8] Sham S. Y. Z., Thambiah S. C., Samsudin I. N., Chuan N. O., Wei Y. S., Razmin N. I. (2016). Practice of self-monitoring blood glucose among insulin-treated diabetic patients in Hospital Serdang. *Malaysian Journal of Medicine and Health Sciences*.

[9] Schnell O., Alawi H., Battelino T. (2013). Self-monitoring of blood glucose in type 2 diabetes: recent studies. *Journal of Diabetes Science and Technology*.

[10] Zhu N. A., Reichert S., Harris S. B. (2020). Limitations of hemoglobin A1c in the management of type 2 diabetes mellitus. *Canadian Family Physician*.

[11] Wang X., Luo J. F., Qi L., Long Q., Guo J., Wang H. H. (2019). Adherence to self-monitoring of blood glucose in Chinese patients with type 2 diabetes: current status and influential factors based on electronic questionnaires. *Patient Preference and Adherence*.

[12] Ji L., Su Q., Feng B. (2017). Structured self-monitoring of blood glucose regimens improve glycemic control in poorly controlled Chinese patients on insulin therapy: results from COMPASS. *Journal of Diabetes*.

[13] Blevins T. (2013). Value and utility of self-monitoring of blood glucose in non-insulin-treated patients with type 2 diabetes mellitus. *Postgraduate Medicine*.

[14] Kambi M., Irunde H., Mbwasi R., Simba A., Nzobo B., Al E. (2001). Standard treatment guidelines and national essential medicines list tanzania mainland. *Government of Tanzania*.

[15] Wambui Charity K., Kumar A. M. V., Hinderaker S. G., Chinnakali P., Pastakia S. D., Kamano J. (2016). Do diabetes mellitus patients adhere to self-monitoring of blood glucose (SMBG) and is this associated with glycemic control? Experiences from a SMBG program in western Kenya. *Diabetes Research and Clinical Practice*.

[16] Franciosi M., Lucisano G., Pellegrini F. (2011). ROSES: role of self-monitoring of blood glucose and intensive education in patients with type 2 diabetes not receiving insulin. A pilot randomized clinical trial. *Diabetic Medicine*.

[17] Schmitt A., Kulzer B., Ehrmann D., Haak T., Hermanns N. (2022). A self-report measure of diabetes self-management for type 1 and type 2 diabetes: the diabetes self-management questionnaire-revised (DSMQ-R) – clinimetric evidence from five studies. *Frontiers in Clinical Diabetes and Healthcare*.

[18] Stetson B., Schlundt D., Rothschild C., Floyd J. E., Rogers W., Mokshagundam S. P. (2011). Development and validation of the personal diabetes questionnaire (PDQ): a measure of diabetes self-care behaviors, perceptions and barriers. *Diabetes Research and Clinical Practice*.

[19] Ng’ang’a L., Ngoga G., Dusabeyezu S. (2022). Feasibility and effectiveness of self-monitoring of blood glucose among insulin-dependent patients with type 2 diabetes: open randomized control trial in three rural districts in Rwanda. *BMC Endocrine Disorders*.

[20] Chubb S. A. P., Van Minnen K., Davis W. A., Bruce D. G., Davis T. M. E. (2011). The relationship between self-monitoring of blood glucose results and glycated haemoglobin in type 2 diabetes: the Fremantle diabetes study. *Diabetes Research and Clinical Practice*.

[21] Mastura H. I., Mimi O., Piterman L., Teng C. L., Wijesinha S. (2007). Self-monitoring of blood glucose among diabetes patients attending government health clinics. *Medical Journal of Malaysia*.

[22] Sodipo O. O., Adedokun A., Adejumo A. (2017). Effect of self-monitoring of blood glucose on glycaemic outcome among type 2 diabetic patients. *South African Family Practice*.

[23] Lalić N. M., Lalić K., Jotić A. (2017). The impact of structured self-monitoring of blood glucose combined with intensive education on HbA1c levels, hospitalizations, and quality-of-life parameters in insulin-treated patients with diabetes at primary care in Serbia: the multicenter SPA-EDU study. *Journal of Diabetes Science and Technology*.

[24] Kato N., Cui J., Kato M. (2013). Structured self-monitoring of blood glucose reduces glycated hemoglobin in insulin-treated diabetes. *Journal of Diabetes Investigation*.

