# Supplementary material for: Impact of Self-Monitoring Blood Glucose on Glycaemic Control Among Insulin-Treated Patients With Diabetes Mellitus in Northeastern Tanzania: A Randomised Controlled Trial
Source: J Diabetes Res. 2024 Jun 10;2024:6789672. doi: 10.1155/2024/6789672 (PMC11186681; doi:10.1155/2024/6789672)
Supplement: Supporting Information — Additional supporting information can be found online in the Supporting Information section. [file 6789672.f1.docx]

**Supplementary Table 1: Association between SMBG and behavioral factors among insulin-treated DM patients seen in the DM clinic at KCMC before intervention**

| **Variable** | **Self-monitoring of blood glucose** | | **p-value** |
| --- | --- | --- | --- |
|  | **Yes, n (%)** | **No, n (%)** |  |
| Years on insulin regimen |  |  | 0.45 |
| <1 | 8 (57.14) | 6 (42.86.) |  |
| 1–5 | 14 (53.85) | 12 (46.15) |  |
| 5–10 | 9 (47.37) | 10 (52.63) |  |
| >10 | 7 (33.33) | 14 (66.67) |  |
| Dosage frequency |  |  | 0.88 |
| Once daily | 2 (66.67) | 1 (33.33) |  |
| Twice daily | 33 (47.14) | 37 (52.86) |  |
| Thrice daily | 3 (42.86) | 4 (57.14) |  |
| Years of diabetes diagnosis |  |  | 0.24 |
| 1-5 years | 4 (66.67) | 2 (33.33) |  |
| 5-10 years | 4 (28.57) | 10 (71.43) |  |
| >10 years | 30 (50) | 30 (50) |  |
| Ever taught on SMBG |  |  | 0.01 |
| Yes | 33 (55.93) | 26 (44.07) |  |
| No | 5 (23.81) | 16 (76.19) |  |
| Do you adjust insulin dose |  |  | 0.5 |
| Yes | 19 (51.35) | 18 (48.65) |  |
| No | 19 (44.19) | 24 (55.81) |  |
| Difficulty to measure BG |  |  | 0.08 |
| Yes | 3 (25) | 9 (75) |  |
| No | 35 (51.47) | 33 (48.51) |  |
| Attended any class extensively |  |  | 0.1 |
| Yes | 29 (53.7) | 25 (46.3) |  |
| No | 9 (34.62) | 17 (65.38) |  |
| Aware of glycemic targets |  |  | 0.06 |
| Yes | 29 (52.7) | 26 (47.3) |  |
| No | 12 (48.0) | 13 (52.0) |  |

Supplementary Table 2: Effect of the intervention on glycemic control (n=80)

| **Variable** | **Crude** |  | **Adjusted** |  |
| --- | --- | --- | --- | --- |
|  | **Coefficient (95% CI)** | ***p*-value** | **Coefficient (95% CI)** | ***p*-value** |
| Group |  |  |  |  |
| Control | Ref |  | Ref |  |
| Intervention | -0.81 (-1.59 – -0.015) | 0.04 | -1.14 (-1.91 – -0.36) | 0.004 |
| Age (years) |  |  |  |  |
| 18–34 | Ref |  |  |  |
| 35–59 | -0.59 (-2.34 – 1.15) | 0.5 |  |  |
| >60 | -0.71 (-2.42 – 1.0) | 0.4 |  |  |
| Educational Status |  |  |  |  |
| Primary Education | Ref |  | Ref |  |
| Secondary and above | 0.85 (0.06 – 1.64) | 0.03 | 1.21 (0.43 – 1.99) | 0.003 |
| Comorbidities |  |  |  |  |
| Hypertension | Ref |  |  |  |
| Chronic kidney disease | 0.009 (-1.88 – 1.91) | 0.9 |  |  |
| Others | 0.06 (-0.81 – 0.95) | 0.87 |  |  |
| BMI |  |  |  |  |
| Normal | Ref |  |  |  |
| Obese/overweight | 0.27 (-0.66 – 1.21) | 0.56 |  |  |
| Years on insulin regimen |  |  |  |  |
| <1 | Ref |  |  |  |
| 1–5 | -0.26 (-1.43 – 0.930) | 0.66 |  |  |
| 6–10 | 0.72 (-0.53 – 1.97) | 0.25 |  |  |
| >10 | 0.65 (-0.57 – 1.880 | 0.29 |  |  |
| Type of Insulin Rx |  |  |  |  |
| Pre mixed insulin | Ref |  |  |  |
| Regular + NPH insulin | -0.016 (-0.94 – 0.913) | 0.9 |  |  |
| Insulin + oral drugs | -0.53 (-1.68 – 0.34) | 0.3 |  |  |
| Total insulin dose | -0.01 (-0.02 – 0.007) | 0.2 | -0.02 (-0.03 – 0.0009) | 0.05 |
